# Significance of bacteria in oviposition and larval development of the sand fly *Lutzomyia longipalpis*

**DOI:** 10.1186/1756-3305-5-145

**Published:** 2012-07-24

**Authors:** Kamila Peterkova-Koci, Maricela Robles-Murguia, Marcelo Ramalho-Ortigao, Ludek Zurek

**Affiliations:** 1 Department of Entomology, Kansas State University, 123 Waters Hall, Manhattan, KS, USA; 2 Department of Diagnostic Medicine and Pathobiology, Kansas State University, 123 Waters Hall, Manhattan, KS, USA

**Keywords:** Sand flies, *Lutzomyia longipalpis*, Bacteria, Oviposition, Larval development, Rabbit feces

## Abstract

**Background:**

Microbial ecology of phlebotomine sand flies is not well understood although bacteria likely play an important role in the sand fly biology and vector capacity for *Leishmania* parasites. In this study, we assessed the significance of the microbial community of rabbit feces in oviposition and larval development of *Lutzomyia longipalpis* as well as bacterial colonization of the gut of freshly emerged flies.

**Methods:**

Sterile (by autoclaving) and non-sterile (control) rabbit feces were used in the two-choice assay to determine their oviposition attractiveness to sand fly females. Bacteria were identified by amplification and sequencing of the 16S rRNA gene with universal eubacterial primers. Sterile, control (non-sterile), and sterilized and inoculated rabbit feces were used to assess the significance of bacteria in *L. longipalpis* development. Newly emerged adult flies were surface-sterilized and screened for the bacterial population size and diversity by the culturing approach. The digestive tract of L4 sterile and control larvae was incubated with Phalloidin to visualize muscle tissues and DAPI to visualize nuclei.

**Results:**

Two-choice behavioural assays revealed a great preference of *L. longipalpis* to lay eggs on rabbit feces with an active complex bacterial community (control) (85.8 % of eggs) in comparison to that of sterile (autoclaved) rabbit feces (14.2 %). Bioassays demonstrated that *L. longipalpis* larvae can develop in sterile rabbit feces although development time to adult stage was greatly extended (47 days) and survival of larvae was significantly lower (77.8 %) compared to that of larvae developing in the control rabbit feces (32 days and 91.7 %). Larval survival on sterilized rabbit feces inoculated with the individual bacterial isolates originating from this substrate varied greatly depending on a bacterial strain. *Rhizobium radiobacter* supported larval development to adult stage into the greatest extent (39 days, 88.0 %) in contrast to that of *Bacillus* spp. (76 days, 36.0 %). From the complex natural bacterial community of rabbit feces, *R. radiobacter* survived pupation and colonized the newly emerged females most successfully (82.6 % of all bacteria cultured); however, only 25 % of females were positive for bacteria in the digestive tract upon emergence. Immunohistochemistry did not reveal any obvious differences in anatomy of the digestive tract between control and axenic larvae.

**Conclusions:**

The bacterial community in the sand fly larval habitat affects oviposition and larval development although bacteria are not essential for successful development of *L. longipalpis*. Different bacteria contribute to larval development to various degrees and some, *e.g. Rhizobium radiobacter,* survive pupation and colonize the digestive tract of newly emerged females. With the establishment of the axenic rearing system, this study opens new venues to study the effect of bacteria on the gut epithelial immunity and vector competence of sand flies for *Leishmania* parasites with a goal to develop paratransgenic approaches for *Leishmania* control.

## Background

The phlebotomine sand fly *Lutzomyia longipalpis* (Diptera: Psychodidae) is an important biological vector of *Leishmania infantum* (syn. *Leishmania chagasi*) in Central and South America. Although very little is known about the larval habitat of sand flies, it is generally agreed that organic material is the main food source for larvae [1]. The larval habitat likely includes feces of rabbits and rodents in underground burrows where the proper temperature and humidity are maintained [2]. Rabbit feces contain a large and diverse microbial community [3,4] that potentially plays an important role in: a) sand fly oviposition behavior as bacterial volatile compounds may be used as semiochemical cues for females to locate the suitable habitat for their offspring; b) sand fly larval development since bacteria may provide essential or additional nutrients; and c) vector competence of sand flies for *Leishmania* parasites as bacteria surviving pupation and colonizing the gut of newly emerged females may influence development and transmission of the parasites.

It has been demonstrated that animal feces, including that of rabbits, play a role in *L. longipalpis* oviposition behavior and the active chemicals for the attraction are hexanal and 2-methyl-2-butanol [5,6]. Additional oviposition attractant and/or stimulant for *L. longipalpis* includes dodecanoic acid deposited on eggs from the female accessory glands [7-9]. Furthermore, frass of the larvae also positively affected *L. longipalpis* oviposition [10]. While the bacterial community in animal feces is likely a major player influencing sand fly oviposition behavior, only one study addressed this topic and showed that bacterial isolates from the soil in the natural breeding habitat of *Phlebotomus papatasi* attracted gravid females [11].

Virtually nothing is known about the significance of bacteria in the larval development of sand flies. It has been shown for several other Diptera including stable flies [12], house flies [13], horn flies [14], and face flies [15] that live bacteria are essential for successful larval development although the basis of this dependence on microbes remains unknown.

While bacteria in the lumen of the digestive tract do not typically survive insect pupation, it has been reported that freshly emerged female mosquitoes *Anopheles gambiae* and *A. stephensi*[16] and sand flies *P. duboscqi*[17] and *P. argentipes*[18] had live bacteria in the digestive tract.

This study was designed to determine the significance of bacteria in rabbit feces in oviposition and larval development of *L. longipalpis* as well as to assess the extent of bacterial survival during sand fly pupation.

## Methods

### Sand flies and rabbit feces

A laboratory colony of *Lutzomyia longipalpis* from Jacobina, Brazil was used in this study. Flies were maintained at 27 ± 1 °C and 75 ± 5 % humidity. Adults were given a 20 % sucrose solution *ad libitum* and females were blood-fed twice a week on anesthetized mouse to produce eggs. For all experiments, fresh (< 24 hrs) rabbit feces from domestic rabbits kept in outdoor pens and fed alfalfa/corn rabbit pellets and grass garden clippings or bromegrass hay, were used for the assays.

### Oviposition assay

In two-choice assays, sterile (by autoclaving) and non-sterile (control) rabbit feces were used to determine their oviposition attractiveness to sand fly females. Individual blood-fed females were placed in the plastic ovipots (ø = 10 cm, height = 7 cm) with plaster on the bottom and 1.0 g of each, sterile and control rabbit feces on opposite sides of the ovipot. Before each assay, humidity of rabbit feces was measured and adjusted to be the same (65-70 %) for both substrates using sterile tap water. Number of eggs on each substrate was recorded after 24 hrs. Nine individual females were used in each bioassay and each assay was replicated five times. At the end of each assay, rabbit feces of each type (control and sterilized) from two ovipots were sampled, diluted in phosphate-buffered saline (PBS) (pH 7.2; MP Biomedicals), and plated on broad spectrum medium, trypticase soy broth agar (TSBA) (BBL, Sparks) to assess the extent of contamination of sterilized feces by the flies during the assay.

### Isolation and identification of bacteria

Fresh (< 24 hrs) rabbit feces were brought to the laboratory in a sterile plastic bag and processed immediately. For the isolation of bacteria, 10 g of feces was suspended in 40 ml of PBS, serially diluted in PBS, and dilutions were plated onto TSBA, and two selective and differentiating media, MacConkey agar (MAC) and modified Enterococcus agar (mENT) (BBL, Sparks). Plates were then incubated aerobically at 26 °C (TSBA), 37 °C (MAC) and 42 °C (mENT) for 48–72 h. Morphologically different single colonies were isolated on TSBA and stored at 4 °C until further analysis. Bacteria were identified by amplification and sequencing of the 16S rRNA gene with universal eubacterial primers: 8F (5'-AGAGTTTGATCC TGGCT CAG-3') and 806R (5'- CTACCAGGGTATCTAAT-3') [19] following the standard protocols. Isolates from mENT (enterococci) were identified by amplification and sequencing of the manganese-dependent superoxide dismutase gene (*sodA*) [20]. Sequences were manually edited in CodonCode Aligner (version 1.3.4) (CodonCode Corporation) and identified by BLAST (Basic Local Alignment Search Tool) [21] search of the GenBank database.

### Larval development assay

Four day old sand fly eggs were surface-sterilized with 0.05 % sodium hypochlorite and 70 % ethanol [13] and placed on TSBA plate until hatching to confirm the surface sterility. Newly hatched larvae (five per plate) were transferred by sterile brush in Petri plates with sterile water agar base. Water agar (1.4 %) was used to maintain appropriate moisture in the plates. For the control group, fresh rabbit feces grounded with mortar and pestle were provided *ad libitum*. For the axenic group, grounded fresh rabbit feces were sterilized by autoclaving and offered *ad libitum*. To assess the contribution of bacteria to larval development, sterile feces were inoculated with different bacterial isolates suspended in PBS (~ 10^6^ CFU per ml). Humidity of each substrate was measured before assays and adjusted by sterile PBS. Plates were kept at 26 °C and 40 ± 5 % humidity until adult emergence. Mortality and development time to pupation and adult emergence were monitored on a daily basis. Each assay was conducted in 5 replicates (inoculated rabbit feces) or in 12 replicates (sterile and control feces). Three plates with sterile feces were discarded due to microbial contamination. Sterility of the substrate in the axenic system was confirmed by plating (100 μl) of the rabbit feces (1 g suspended in 10 ml PBS) on TSBA at the end of the assay. To assess the fitness of adult flies from the axenic group, they were fed sterile 20 % sucrose solution offered on sterilized filter papers for 3 days.

### Survival of bacteria during sand fly pupation

Newly emerged flies were surface-sterilized (as described above), homogenized by hand using pestles in 100 μl PBS and plated on TSBA maintained at 26 °C under aerobic conditions. Bacterial counts (CFU per fly) were determined and bacterial colonies with distinct morphologies were sub-cultured and identified by amplification and sequencing of the 16S rRNA gene as described above. Sterility of axenic females was confirmed by plating the fly homogenate on TSBA and by DNA extraction and amplification of 16S rDNA with universal primers as described above.

### Gut immunohistochemistry

The digestive tract of L4 larvae was dissected in PBS and incubated for 10 min in dark with Alexa Fluor 546 Phalloidin (Molecular Probes) to visualize muscle tissues (Actin) and DAPI (4', 6-diamidino-2-phenylindole) (Sigma) to visualize nuclei. Samples were observed under the compound microscope (Nikon Eclipse E800) with epifluorescent UV light and appropriate filters. Four individual digestive tracts from each group (control and axenic) were analyzed.

### Statistical analysis

One-way ANOVA test was used to assess significance of differences in mortality and development time among flies in different treatments using Origin 7 (OriginLab Corp.). If ANOVA revealed significant differences (*P* ≤ 0.05) among treatments, pairwise comparisons were conducted using Tukey test in Origin 7 to assign groupings.

## Results and Discussion

**Figure 1:**
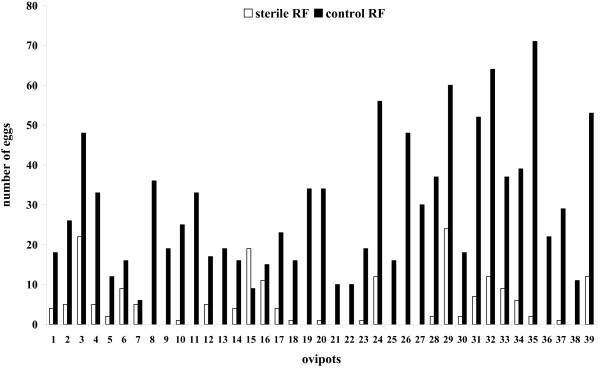
**Sand fly oviposition preference for the control and sterile rabbit feces (RF).** Thirty-nine ovipots (X axis) containing sterile and non-sterile rabbit feces were used for the analyses.

Analysis of oviposition response of sand fly females to sterile and control rabbit feces showed a clear preference to feces with a live and complex microbial community (Figure 1). The majority of eggs (85.8 %) were laid on or very near control rabbit feces (29.2 ± 16.9 eggs per female) in comparison to sterile feces where only 14.2 % of eggs were oviposited (4.8 ± 6.2 eggs per female). Although the sterilized feces did not remain sterile at the end of the assay (after 24 hrs) due to contamination by the sand flies, the difference in concentration was obvious (~ 10^6^ CFU per gram [control] *versus* ~10^2^ CFU per gram [sterilized]). These data clearly show that the live bacterial community in rabbit feces produces chemical cues that serve as attractant and/or stimulant for oviposition of *L. longipalpis*. Whether these cues are volatile or serve as contact semiochemicals needs to be assessed in future studies. Previously, two compounds originating from rabbit feces reported as oviposition attractants were hexanal and 2-methyl-2-butanol [22]. Hexanal is a bi-product of lipid oxidation and not likely of the microbial origin. On the other hand, 2-methyl-2-butanol, is generated by microbial fermentation supporting the notion that the microbial community generates oviposition attractants or stimulants. Nevertheless, the key bacterial taxa and their metabolic products affecting sand fly oviposition behavior remain to be determined. Autoclaving not only sterilized the substrate and killed the microbes but likely affected biological and chemical properties of the rabbit feces and consequently volatile compounds. However, the sterilized feces were not used for oviposition assays for 24 hrs after autoclaving in order to cool down and allow for exchange of the headspace air. Our previous study [12] using the same approach to assess the significance of bacteria in horse feces in oviposition of stable flies, demonstrated that inoculation of autoclaved feces with bacterial isolates restored the oviposition attractiveness of this substrate. While this remains to be shown for sand flies, it supports the notion that it is bacterial volatiles that are used as cues for insect oviposition.

The following seventeen bacterial isolates from fresh rabbit feces with different colony morphology were identified to the species or genus level by sequencing of the 16S rRNA gene (655–698 bp) or the *sodA* gene (438 bp): *Bacillus firmus* (98 % identity), *Bacillus* sp. KK-1 (99 %); *Enterococcus avium* (99 %); *Enterococcus gallinarum* (99 %); *Curtobacteriun flaccumfaciens* (99 %); *Microbacterium foliorum* (98 %), *Arthrobacter bergeri* (99 %), *Arthrobacter* sp. (99 %), *Acinetobacter* sp. (99 %); *Citrobacter freundii* (99 %), *Morganella morganii* (99 %), *Escherichia coli* (99 %)*, Klebsiella* sp. (99 %), *Enterobacter* sp. (97 %), *Pseudomonas* sp. KK-1 (99 %), *Pseudomonas* sp. KK-2 (99 %), and *Rhizobium radiobacter* (100 %). Analysis of the isolates from the digestive tract of freshly emerged sand fly females that developed as larvae in control rabbit feces led to identification of two additional bacterial taxa, *Mycobacterium phocaicum* (99 %) and *Acidovorax* sp. (99 %). Most of these bacteria are common members of the mammalian gastro-intestinal tract and some likely originate from the rabbit environment (soil) and feed (grass clippings and hay). Several bacterial species reported in our study including *Pseudomonas, Citrobacter, Enterobacter, Escherichia, Klebsiella,* and *Morganella* sp. were detected previously by culturing approach in the midgut of wild *L. longipalpis* collected from three sites in Brazil [23]. A similar bacterial community was also found by culturing of the gut bacteria from *P. argentipes* in India [24] although in this study, higher prevalence of Gram-positive taxa (*Bacillus, Staphylococcus, Micrococcu*s sp.) was found compared to that of Gouveia *et al.*[23].

**Figure 2:**
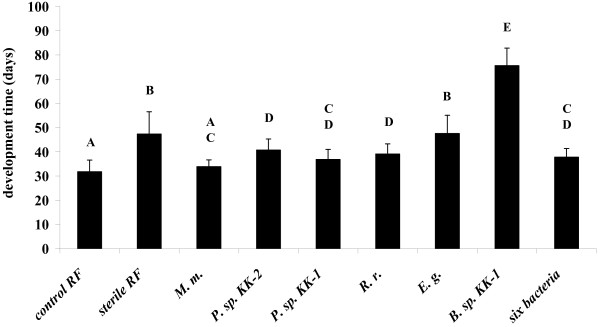
**Development time (mean + SEM) of sand flies reared on sterile rabbit feces inoculated with various bacterial isolates.** RF rabbit feces; *M. m. - Morganella morganii; P. sp.* KK-2 *- Pseudomonas sp.* KK-2; *P. sp.* KK-1 *- Pseudomonas sp.* KK-1; *R .r. - Rhizobium radiobacter; E. g. - Enterococcus gallinarum; B. sp.* KK-1 *- Bacillus sp.* KK-1. Different letters above error bars indicate significant differences (*P* ≤ 0.05).

Mortality (22.2 %) was significantly higher and development time to adult stage (47.4 ± 9.1 days) was significantly longer in larvae maintained in axenic (sterile) rabbit feces compared to that of larvae in control rabbit feces with a live and complex microbial community (8.3 % mortality, 31.8 ± 4.8 days development time) (Table 1, Figure 2). Six bacterial isolates identified from fresh rabbit feces were selected for larval developmental assay to evaluate their significance in *L. longipalpis* development individually and in combination. Several individual bacterial isolates including *Pseudomonas* sp. KK-1, *Rhizobium radiobacter*, *Enterococcus gallinarum*, and all six isolates together supported development of *L. longipalpis* larvae to adult stage into the similar extent (mortality not significantly different) as the entire microbial community (control rabbit feces) (Table 1). Mortality of larvae grown on rabbit feces with *Morganella morganii* and *Pseudomonas* sp. KK-2 was significantly higher than that on control rabbit feces (Table 1). From the development time perspective (1^st^ instar to adult stage), among all individual isolates, feces with *M. morganii* led to the shortest development time (33.9 ± 2.8 days) not significantly different from that of control rabbit feces. In contrast, development with *E. gallinarum* was long and not significantly different from that of sterile feces (Figure 2). *Bacillus* sp. KK-1 supported the development of sand fly larvae the least, resulting in very high mortality (64 %) and significantly longer development time (75.6 ± 7.2 days) compared to that of all other treatments (Table 1, Figure 2).

**Table 1:** Development of *L. longipalpis* in rabbit feces (RF) with different treatments and prevalence and concentration of bacteria in the gut of newly emerged females

**Substrate**	**1**^**st**^**instar larvae (n)**	**% survival to adult stage (% females)**	**% newly emerged females with bacteria in the gut**	**Mean concentration of bacteria (CFU ± SEM) per female**^**#**^
Control RF	60	91.7 (50.9)^a^	25.0	1.4 ± 0.9 × 10^1^
Sterile RF	45	77.8 (48.6)^b^	0	0
*Morganella morgani*	25	76.0 (52.6)^b^	40.0	1.6 ± 1.5 × 10^3^
*Pseudomonas sp.* KK-2	25	72.0 (55.6)^b^	40.0	3.2 ± 2.6 × 10^2^
*Pseudomonas sp.* KK-1	25	84.0 (38.1)^a^	25.0	2.0 ± 1.2 × 10^3^
*Rhizobacterium radiobacter*	25	88.0 (40.9)^a^	33.3	1.8 ± 1.3 × 10^3^
*Enterococcus gallinarum*	25	84.0 (76.2)^a^	6.3	8.3 × 10^1^
*Bacillus sp.* KK-1	25	36.0 (22.2)^c^	0	0
six bacterial isolates together	25	84.0 (42.9)^a^	44.4	2.8 ± 2.3 × 10^2*^

CFU = colony forming units; different letters in superscript indicate significant difference among treatments.

**Figure 3:**
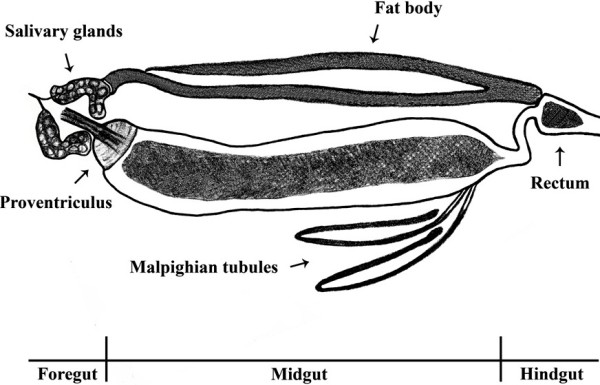
Schematic drawing of L4 larva digestive tract, salivary glands, fat body, and Malpighian tubules.

^#^ only females positive for bacteria are included, SEM = standard error of mean, *bacterial diversity shown in Figure 3.

The basis of bacterial contribution to sand fly larval development is unknown; it may include breakdown of the components of rabbit feces to more digestible and absorbable nutrients and/or production of additional nutrients such as vitamins and amino acids. Although bacterial cells may be digestible in the gut of sand fly larvae and may serve as a source of nutrients, the bacterial cells mass alone (no additional food sources) does not support larval development (data not shown). It is important to emphasize that this study focused on the bacterial community only and other microorganisms including fungi and protozoa may also play a role in sand fly oviposition and larval development.

**Figure 4:**
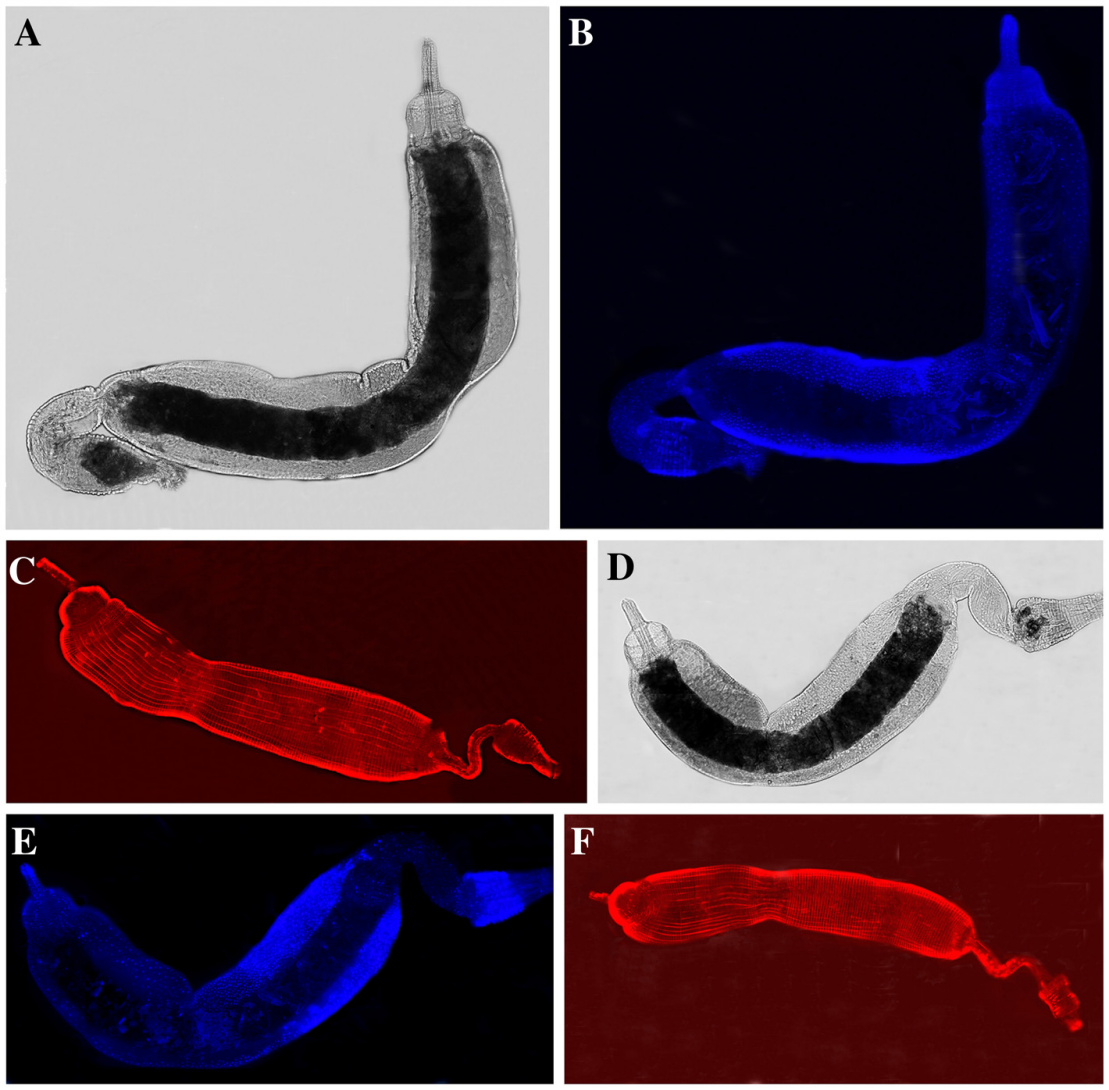
Immunohistochemistry of the larval (L4) digestive tract: Control larva: (A1) regular light, (A2) DAPI staining, (A3) phalloidin staining; Axenic larva: (B1) regular light, (B2) DAPI staining, (B3) phalloidin staining.

To our knowledge, the organization of the internal organs of sand fly larvae has not been reported with the exception of the study by Fazio do Vale *et al.*[25], showing anatomy of the gut of *L. longipalpis* and where pH gradient and several proteinases in the midgut were measured. Here we show the L4 digestive tract, salivary glands, fat body (one side only), and Malpighian tubules (one side only) in the drawing based on our microscopy (Figure 3). We did not observe any obvious differences in the gut size between axenic and control larvae. In addition, our preliminary observation of the gut structure including muscle tissue (actin staining with phalloidin) and epithelium (nuclei staining with DAPI) (Figure 4) did not indicate any obvious differences between axenic and control larvae. However, this remains to be examined in more details. We also did not detect any noticeable differences between axenic and control adult sand flies in regards to body size, sugar feeding, and mortality. Since we dissected axenic adult flies either immediately after emergence or after 3 days of sucrose feeding to confirm sterility of the digestive tract, overall adult life span, blood feeding, and fecundity of axenic females are unknown and will be addressed in our future studies.

**Figure 5:**
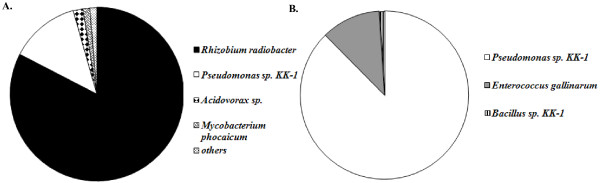
Diversity and prevalence of bacteria in the digestive tract of newly emerged sand flies that developed in control (A) and sterile rabbit feces inoculated with a mixture of six bacterial isolates (B).

Newly emerged females that developed as larvae in control rabbit feces with an active and complex bacterial community had live bacteria in the digestive tract although the prevalence was low (25 % of flies) and the bacterial community size (8.3 × 10^1^ to 2.0 × 10^3^ CFU per gut) and diversity were also low (Table 1, Figure 5A). Interestingly, from the complex bacterial community of control feces, two bacterial species, *Rhizobium radiobacter* and *Pseudomonas* sp. KK-1, that supported sand fly development to a great extent, also survived pupation and colonized the gut of females most commonly (95.6 % of all isolates) (Figure 5A). Survival of individual bacterial species during fly pupation varied greatly depending on the bacterial strain; *M. morganii* and *Pseudomonas* sp. KK-2 were detected in 40 % of females while *Bacillus* sp. KK-1 was not detectable although due to high larval mortality only two adult females were analyzed (Table 1). The highest prevalence of bacteria in the gut of newly emerged females (44.4 %) was detected from the larval substrate inoculated by the mixture of all six isolates. The highest population size (~10^3^ CFU) per gut was recorded for *Rhizobium radiobacter* and *Pseudomonas* sp. KK-1 (Table 1). In contrast to results from the complex microbial community, *R. radiobacter* was not recovered and *Pseudomonas* sp. KK-1 and *E. gallinarum* were two most frequently detected bacterial taxa in the gut of newly emerged females that developed as larvae in a substrate with the mixture of six isolates (Figure 5B). Sterility of axenic flies (from sterile rabbit feces) was confirmed by a culturing method as well as by PCR of 16S rDNA.

In general, before and during pupation, bacteria in the gut lumen are eliminated due to major changes in the gut content and structure and secretion of antimicrobial compounds [26]. However, transstadial passage of bacteria from larvae to pupae and adult flies has been reported for *P. duboscqi*[17] and *P. argentipes*[18]. In *P. duboscqiOchrobactrum* sp. AK was recovered in the midgut and hindgut of 64 % of freshly emerged flies and their counts ranged from (10^2^ to 10^4^ CFU per fly). Hurwitz *et al.*[18] showed that *B. subtilis* added (10^7^ CFU) to the *P. argentipes* (L4) larval medium (fermented rabbit food and feces) either sterilized or control (with other live bacteria) survived pupation and was recovered in 75 % of flies in concentration 7.2 x 10^3^ CFU per fly (sterilized medium) and 74 % of flies with 3.9 x 10^4^ CFU per fly (control medium). The prevalence and concentration of bacteria in those two studies was higher compared to ours; this may be due to differences in survivability between bacteria and also differences in the gut structure and pupation between the sand fly species. Furthermore, Hurwitz *et al.*[18] reported that *P. argentipes* failed to develop in a sterile substrate, which is in contrast to our study with *L. longipalpis*. However, so far, we have not been able to raise *P. papatasi* on sterile rabbit feces (data not shown) and it may be that there is a difference in dependence on bacteria between Old World and New World sand fly species. Preference of *Phlebotomus* sp. larvae for non-autoclaved food is also indicated in the study of Volf and Volfova [27] although more data on the significance of microbes in larval development of sand flies in this genus are needed.

## Conclusions

Females of the phlebotomine sand fly *L. longipalpis* use bacteria-mediated cues to locate an appropriate oviposition substrate. In addition, although bacteria contribute to larval development, an axenic system to raise *L. longipalpis* is reported. Some bacteria such as *R. radiobacter* and *Pseudomonas* sp. KK-1 support larval development to the same extent as a complex bacterial community, survive fly pupation, and colonize the digestive tract of newly emerged females. With the establishment of the axenic rearing system, this study opens new venues to study the effect of bacteria on the gut epithelial immunity and vector competence of sand flies for *Leishmania* parasites.

## Competing interests

The authors declare that they have no competing interests.

## Authors' contributions

LZ, KPK, MRO designed the study. KPK conducted the experiments; KPK and LZ analyzed the data and wrote the manuscript. MRM maintained the laboratory colony of *L. longipalpis* and provided blood-fed females for the oviposition assays. All authors read and approved the final manuscript.
